# Association between HALP (hemoglobin, albumin, lymphocyte, and platelet) score and poor outcomes in acute ischemic stroke patients with type 2 diabetes mellitus: a study from the Third China National Stroke Registry

**DOI:** 10.3389/fneur.2024.1461188

**Published:** 2025-01-07

**Authors:** Xu Zhu, Yijun Zhang, Anxin Wang, Xiaoli Zhang, Guoyuan Yu, Shifeng Xiang, Yiping Wu, Xia Meng

**Affiliations:** ^1^Department of Neurology, Handan Central Hospital, Handan, China; ^2^Department of Neurology, Beijing Tiantan Hospital, Capital Medical University, Beijing, China; ^3^China National Clinical Research Center for Neurological Diseases, Beijing Tiantan Hospital, Capital Medical University, Beijing, China; ^4^Department of Epidemiology, Beijing Neurosurgical Institute, Beijing Tiantan Hospital, Capital Medical University, Beijing, China; ^5^Department of Clinical Epidemiology and Clinical Trial, Capital Medical University, Beijing, China; ^6^Beijing Municipal Key Laboratory of Clinical Epidemiology, Beijing, China

**Keywords:** acute ischemic stroke, type 2 diabetes mellitus, HALP scores, outcome, stroke

## Abstract

**Background:**

The combined index (HALP) of hemoglobin, albumin, lymphocytes, and platelets is considered a novel scoring system that reflects systemic inflammation and nutritional status. This study aimed to investigate the relationship between HALP scores and poor outcomes in acute ischemic stroke (AIS) patients with type 2 diabetes mellitus (DM).

**Methods:**

Patients with AIS and type 2 DM were screened from the Third China National Stroke Registry (CNSR-III) and divided into quartiles based on their HALP scores at admission. Clinical outcomes were adverse functional outcomes (modified Rankin scale [mRS] score of 3–6 or 2–6) and all-cause mortality at 3 months and 1 year. The association of HALP with the risk of poor functional outcome and all-cause mortality were analyzed by multivariable logistic regression and Cox proportional hazards regression.

**Results:**

A total of 3,603 patients were included in this study. After adjusting for confounders, it was found that patients in the highest HALP score quartile had lower mRS scores of 2–6 (odds ratio [OR], 0.64; 95% confidence interval [CI], 0.51–0.80) and 3–6 (OR, 0.53; 95% CI, 0.51–0.82) at the 3-month follow-up. At the 1-year follow-up, a significant correlation was observed between HALP scores and mRS scores of 2–6 (OR, 0.65; 95%CI, 0.57–0.81) and 3–6 (OR, 0.64; 95%CI, 0.47–0.86). Additionally, the highest HALP score quartile was associated with a reduced risk of all-cause mortality at the 3-month follow-up (hazard ratio [HR], 0.35; 95%CI, 0.13–0.93). Similar results were observed at the 1-year follow-up (HR, 0.34; 95%CI, 0.18–0.63).

**Conclusion:**

At 3 months of AIS patients with type 2 diabetes and 1-year follow-up, lower HALP scores were associated with poorer functional outcomes and all-cause mortality.

## Introduction

Acute ischemic stroke (AIS) is the main cause of mortality and disability worldwide and significantly increases global health-care spending (1). Despite good medical care, nearly 40% of patients with AIS have a poor prognosis, so we need to identify more factors that affect prognosis. Epidemiological studies have established diabetes mellitus (DM) as a significant and independent risk factor for vascular health (2, 3). Patients with diabetes face a 2.5–3.5 times higher risk of experiencing a stroke compared to those without diabetes (4). A large study analyzed 838,000 patients with AIS in 1,476 centers of the Chinese Stroke Center Alliance from 2012 to 2019 revealing significant findings that 34.2% of AIS patients were combined with or possibly with diabetes (5). Therefore, stroke patients with diabetes have become a special group of concern.

Anemia, malnutrition, and inflammation play significant roles in the progression and prognosis of AIS. Patients with AIS who develop anemia during hospitalization have a higher risk of mortality, additional cardiovascular diseases, comorbidities, and poorer prognoses (6). Albumin (ALB), a marker of human nutritional status, has been extensively utilized in prognostic studies of cerebrovascular disease. A multicentre study of ischemic stroke in China demonstrated an independent association between reduced serum albumin levels and poor prognosis (7). Inflammatory and immune mechanisms play a crucial role in the development of ischaemic stroke, and the presence of lymphocytes has been observed in the aged brain, correlating with a poor prognosis (8, 9). Platelet hyperfunction elevates the risk of cerebrovascular embolism and lesions (10), and also closely linked to the process of acute and chronic inflammation (11). Furthermore, AIS patients with type 2 DM tend to be more susceptible to anemia, malnutrition, and inflammation during hospitalization. Consequently, a scoring system incorporating multidimensional indicators may be more suitable for the prognosis evaluation of stroke patients with diabetes. In recent years, the combined hemoglobin-albumin lymphocyte and platelet scoring system (HALP) has emerged as a marker of systemic inflammation and nutritional status and has been widely used in the evaluation of multiple tumors and cardiovascular diseases (12–14). However, little attention has been focused on the association between HALP score and the risk of poor prognosis and all-cause mortality in this special group of patients who had AIS with type 2 DM.

In the last few years, despite the emergence of HALP in the literature as a new prognostic biomarker for a variety of diseases, however, it was not clear whether HALP scores were associated with poor prognosis in patients with AIS and type 2 DM. To address this issue, we conducted the study to assess the correlation of HALP score with prognosis and all-cause mortality in patients with AIS and type 2 DM.

## Methods

### Study population

The data for this research were derived from the Third China National Stroke Registry (CNSR-III). The dataset included 15,166 patients with acute ischemic cerebrovascular events, recorded from August 2015 to March 2018 at 201 sites in China. All patients were 18 years of age or older and were diagnosed within 7 days of the onset as AIS or transient ischemic attack (TIA). Details, rationale, and a comprehensive description of the CNSR-III were documented previously (15). This research was conducted in strict compliance with the Declaration of Helsinki and received approval from the Ethics Committee of Beijing Tiantan Hospital (IRB approval number: KY2015-001-01).

### Baseline data collection

Research coordinators at each site proactively collected baseline data through face-to-face interviews or by reviewing medical records. The data included demographic characteristics (age, gender, marital status, education level, heavy drinking, current smoking), physical examination [body mass index (BMI), calculated as weight in kilograms divided by the square of height in meters, kg/m^2^], systolic blood pressure (SBP), heart rate at admission, National Institutes of Health Stroke Scale (NIHSS) score at admission, medical history [AIS or TIA, intracerebral hemorrhage (ICH), hypertension, coronary heart disease, dyslipidemia], the cause of AIS classified according to the Trial of Org 10,172 in Acute Stroke Treatment (TOAST) criteria, in-hospital treatment [tissue plasminogen activator (TPA), mechanical thrombectomy (MT), antiplatelet, anticoagulant, antihypertensive, antihyperlipidemic, traditional Chinese medicine (TCM)], and laboratory values [hemoglobin (HGB), lymphocyte count (LY), platelet count (PLT), albumin (ALB)].

### Sample collection and definition of HALP score

Fasting blood samples, including measurements of serum albumin, hemoglobin, lymphocyte count, and platelet count, were obtained within 24 h of admission following stroke onset. The HALP score was calculated using the formula: Hemoglobin (g/L) × Albumin (g/L) × Lymphocyte count (10^9^/L)/Platelet count (10^9^/L). HALP scores were categorized into four quartile-based groups: <35.32, 35.32–47.63, 47.63–64.21, and >64.21.

### Sample size

Currently, events per variable (EPV) criteria, specifically 10 EPV, are widely used as a method of estimating sample size (16). A total of 23 potential confounding variables for adjusted were included in this study. Therefore, 10 EPV were 230 patients. The incidence of poor prognosis in AIS patients is about 14% (17), and the sample size should be at least 1,500 patients. A total of 3,603 patients were included in this study, which can fully meet statistical needs.

### Follow-up and outcome assessment

Functional outcomes were defined using the modified Rankin Scale (mRS) score, a measure of disability widely used to assess recovery after stroke; the primary outcome was mRS 3–6 at 3 months and 1 year, and the secondary outcomes were MRS 2–6 and all-cause mortality at 3 months and 1 year, which were considered poor outcomes. Patients included in the study were followed up at 3 months and 1 year after the onset of the disease by trained study coordinators through face-to-face or telephone interviews. Mortality information was obtained from relatives and verified through death certificates from attended hospitals or local civil registries. Mortality included deaths from any cause.

### Statistical analysis

Continuous variables were expressed as median with interquartile range (IQR); categorical variables were expressed as frequencies (*n*) and percentages (%). Tests for linear trend of baseline characteristics across the HALP score quartiles were performed using Kruskal–Wallis tests for continuous variables and *χ*^2^ trend analysis for categorical variables. One-way and corrected logistic and cox models were used to estimate the relationship between exposure and outcome, with effect values expressed as odds ratios (OR)/hazard ratios (HR) and their 95% confidence intervals (CI). Variables adjusted in multivariable models were age and gender, SBP, BMI, NIHSS score at admission, drinking and smoking, TOAST, medical history (AIS or TIA, ICH, hypertension, coronary heart disease, dyslipidemia), in-hospital treatment (TPA, MT, antiplatelet, anticoagulant, antihypertensive, antihyperlipidemic, TCM). Furthermore, restricted cubic spline was used to explore relationships between HALP score and risk of poor outcomes and all-cause mortality. Stratified analyses were performed based on age (<65 or ≥65 years), sex (male or female), BMI (≤25 or >25 kg/m^2^), and current smoking (yes or no). We applied the likelihood ratio test to assess the interaction significance between stratification variables and the HALP score. Receiver operating characteristic (ROC) curves were cited to evaluate the prognostic value of the HALP score by calculating *p*-values and the area under the curve (AUC). A *p*-value of less than 0.05 (two-sided) was considered highly significant. All statistical analyses were performed using SAS 9.4 software (SAS Institute, Inc., Cary, NC).

## Results

### Baseline characteristics

**Figure 1:**
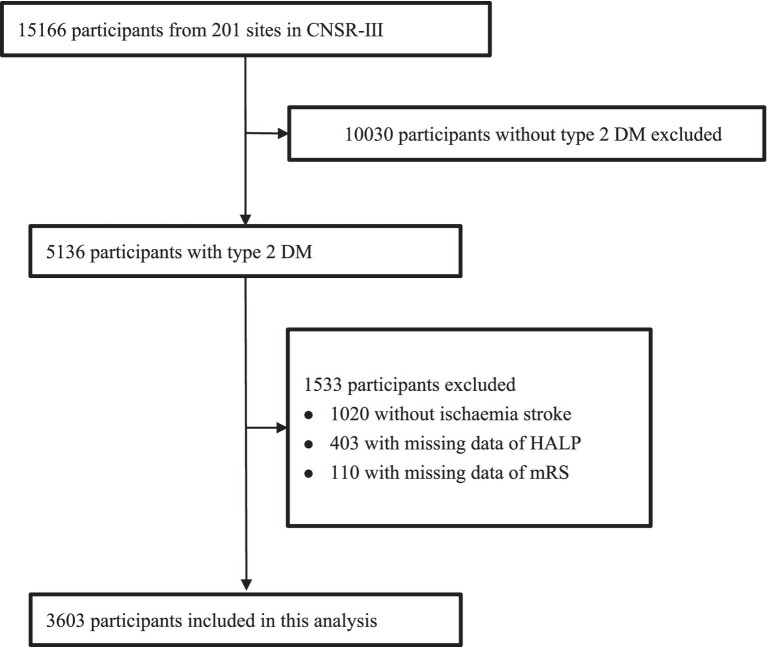
Flow chart of sample selection and the exclusion criteria. CNSR-III, the Third China National Stroke Registry; DM, diabetes mellitus.

Our study involved 5,136 AIS patients with type 2 diabetes from CNSR-III study. 1,020 patients diagnosed with TIA were excluded. 403 patients were also excluded due to lack of HALP data as well as 110 patients who were lost to follow-up. Ultimately, we included 3,603 patients in this study (Figure 1). Table 1 shows the baseline characteristics of the different groups of participants. Compared with the rest of the quartiles, patients in the first quartile of HALP score tended to be older, male, higher NIHSS scores, higher SBP, higher PLT levels, and lower levels of HGB, LY, and ALB.

**Table 1:** Baseline characteristics of patients according to HALP score quartiles.

Variable	Total (*n* = 3,603)	HALP score				P value
		Q1 (<35.32)	Q2 (35.32–47.63)	Q3 (47.63–64.21)	Q4 (>64.21)	
Age, years, median (IQR)	63.00 (56.00–70.00)	65.00 (58.00–72.00)	63.00 (55.00–70.00)	62.00 (55.00–69.00)	61.00 (54.00–68.00)	<0.001
Male, *n* (%)	2,279 (63.25)	454 (50.44)	547 (60.71)	584 (64.82)	694 (77.03)	<0.001
Marital, *n* (%)						0.025
Married	3,396 (94.25)	831 (92.33)	849 (94.23)	857 (95.12)	859 (95.34)	
Others	207 (5.75)	69 (7.67)	52 (5.77)	44 (4.88)	42 (4.66)	
BMI, kg/m^2^, median (IQR)	24.97 (23.12–27.04)	24.49 (22.49–26.73)	24.91 (23.03–26.68)	25.15 (23.44–27.34)	25.22 (23.44–27.34)	<0.001
SBP, mmHg, median (IQR)	150.00 (137.50–165.00)	152.50 (140.00–169.00)	150.00 (138.50–164.00)	150.00 (136.00–165.00)	150.00 (135.50–164.00)	0.003
Heart rate, median (IQR)	76.00 (70.00–82.00)	76.00 (70.00–82.00)	76.00 (70.00–82.00)	76.00 (70.00–82.00)	76.00 (70.00–82.00)	0.434
NIHSS score, median (IQR)	3.00 (2.00–6.00)	4.00 (2.00–7.00)	4.00 (2.00–6.00)	3.00 (2.00–6.00)	3.00 (1.00–5.00)	<0.001
Education, *n* (%)						0.100
Middle school or below	3,278 (91.98)	835 (92.78)	822 (91.23)	814 (90.34)	807 (89.57)	
High school or above	325 (9.02)	65 (7.22)	79 (8.77)	87 (9.66)	94 (10.43)	
Heavy drinking, *n* (%)	459 (12.74)	71 (7.89)	123 (13.65)	113 (12.54)	152 (16.87)	<0.001
Current smoking, *n* (%)	985 (27.34)	158 (17.56)	216 (23.97)	272 (30.19)	339 (37.62)	<0.001
Medical history, *n* (%)						
AIS or TIA	933 (25.90)	254 (28.22)	233 (25.86)	238 (26.42)	208 (23.09)	0.095
ICH	55 (1.53)	7 (0.78)	18 (2.00)	19 (2.11)	11 (1.22)	0.064
Hypertension	2,515 (69.80)	659 (73.22)	627 (69.59)	615 (68.26)	614 (68.15)	0.066
Coronary heart disease	569 (15.79)	162 (18.00)	133 (14.76)	130 (14.43)	144 (15.98)	0.151
Dyslipidemia	408 (11.32)	98 (10.89)	96 (10.65)	111 (12.32)	103 (11.43)	0.689
TOAST types, *n* (%)						0.004
LAA	1,042 (28.92)	262 (29.11)	283 (31.41)	274 (30.41)	223 (24.75)	
CE	171 (4.75)	48 (5.33)	42 (4.66)	42 (4.66)	39 (4.33)	
SAO	836 (23.20)	177 (19.67)	196 (21.75)	221 (24.53)	242 (26.86)	
Others	1,554 (43.13)	413 (45.89)	380 (42.18)	364 (40.40)	397 (44.06)	
In-hospital treatment, *n* (%)						
TPA	240 (6.66)	59 (6.56)	47 (5.22)	80 (8.88)	59 (5.99)	0.013
MT	7 (0.19)	3 (0.33)	1 (0.11)	3 (0.33)	0 (0.00)	0.276
Antiplatelet	3,508 (97.36)	873 (97.00)	885 (98.22)	879 (97.56)	871 (96.67)	0.181
Anticoagulant	352 (9.77)	98 (10.89)	83 (9.21)	79 (8.77)	92 (10.21)	0.420
Antihypertensive	1890 (52.45)	496 (55.11)	449 (49.83)	464 (51.50)	481 (53.39)	0.129
Antihyperlipidem	3,475 (96.45)	866 (96.22)	871 (96.67)	867 (96.23)	871 (96.67)	0.914
TCM	2,150 (59.67)	536 (59.56)	549 (60.93)	529 (58.71)	536 (59.49)	0.812
Laboratory test						
HALP score, median (IQR)	47.63 (35.32–64.21)	28.51 (22.72–31.92)	41.19 (38.30–44.49)	55.11 (51.27–59.17)	78.63 (70.50–93.15)	<0.001
HGB, g/L, median (IQR)	141.00 (130.00–151.00)	130.00 (119.00–140.00)	139.00 (130.00–149.00)	143.00 (132.00–152.00)	149.00 (140.00–159.00)	<0.001
LY, ×10^9^/L, median (IQR)	1.76 (1.38–2.25)	1.29 (1.01–1.53)	1.64 (1.36–1.94)	1.97 (1.60–2.32)	2.35 (1.95–2.80)	<0.001
PLT, ×10^9^/L, median (IQR)	210.00 (174.00–253.00)	239.00 (198.00–282.00)	222.00 (189.00–264.00)	207.00 (173.00–244.00)	181.00 (148.00–213.00)	<0.001
ALB, g/L, median (IQR)	40.43 (38.00–43.00)	39.00 (36.20–41.40)	40.10 (38.00–42.60)	40.80 (38.40–43.10)	41.80 (39.70–44.10)	<0.001

HALP, hemoglobin-albumin lymphocyte, and platelet scoring system; SD, standard deviation; IQR, interquartile range; BMI, body mass index; SBP, systolic blood pressure; NIHSS, National Institute of Health Stroke Scale; mRS, modified Rankin Scale; AlS, acute ischemic stroke; TlA, transient ischemic attack; ICH, intracranial hemorrhage; TOAST, the Trail of Org 10,172 in Acute Stroke Treatment; LAA, large artery atherosclerosis; CE, cardioembolism; SAO, small artery occlusion; TPA, tissue plasminogen activator; MT, mechanical thrombectomy; TCM, traditional Chinese medicine; HGB, hemoglobin; LY, lymphocyte count; PLT, platelet count; ALB, albumin.

### Relationship between HALP score and poor outcome

**Figure 2:**
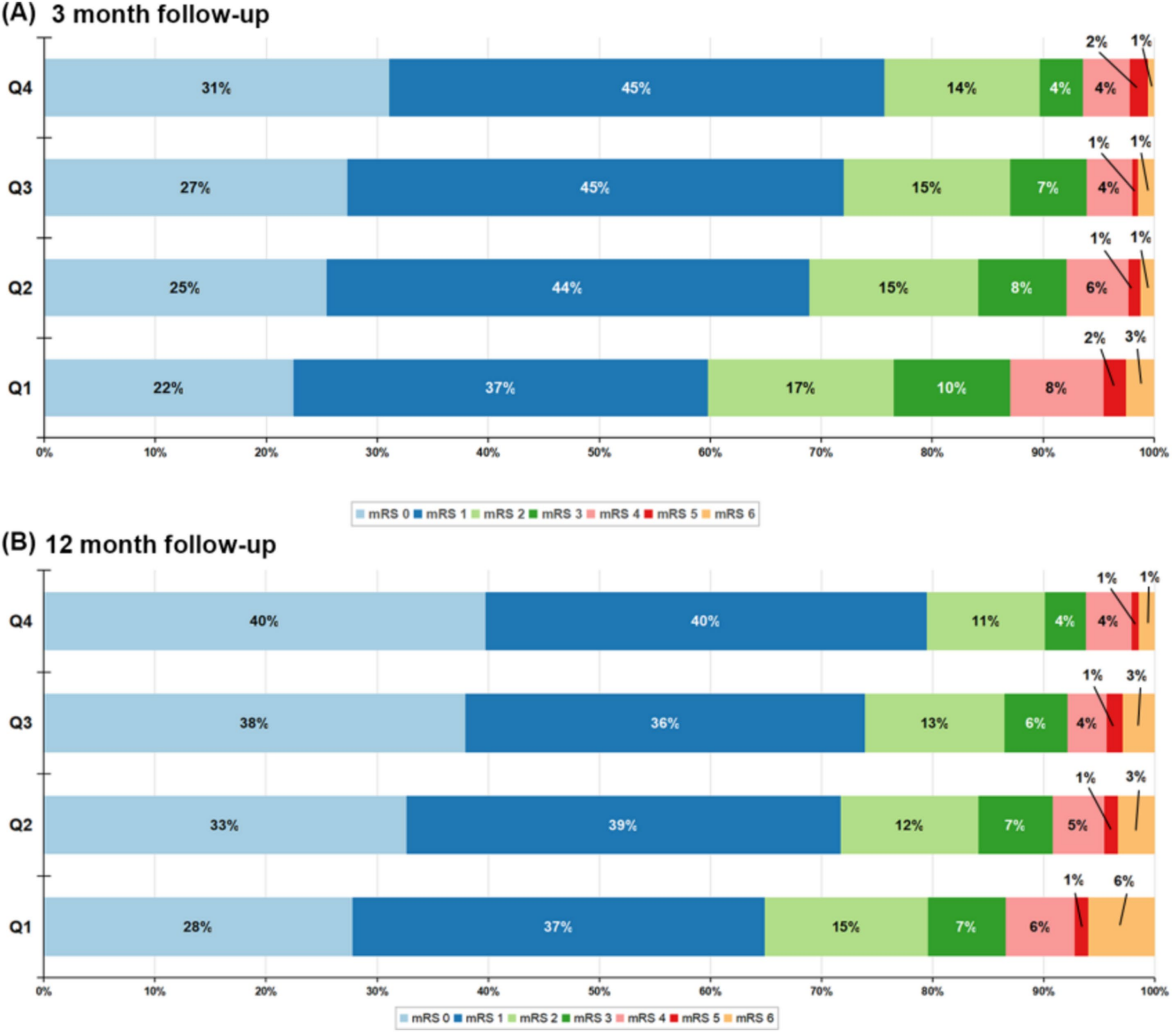
Distribution of mRS score at 3 months **(A)** and 12 months **(B)**. mRS, modified Rankin Scale.

**Figure 3:**
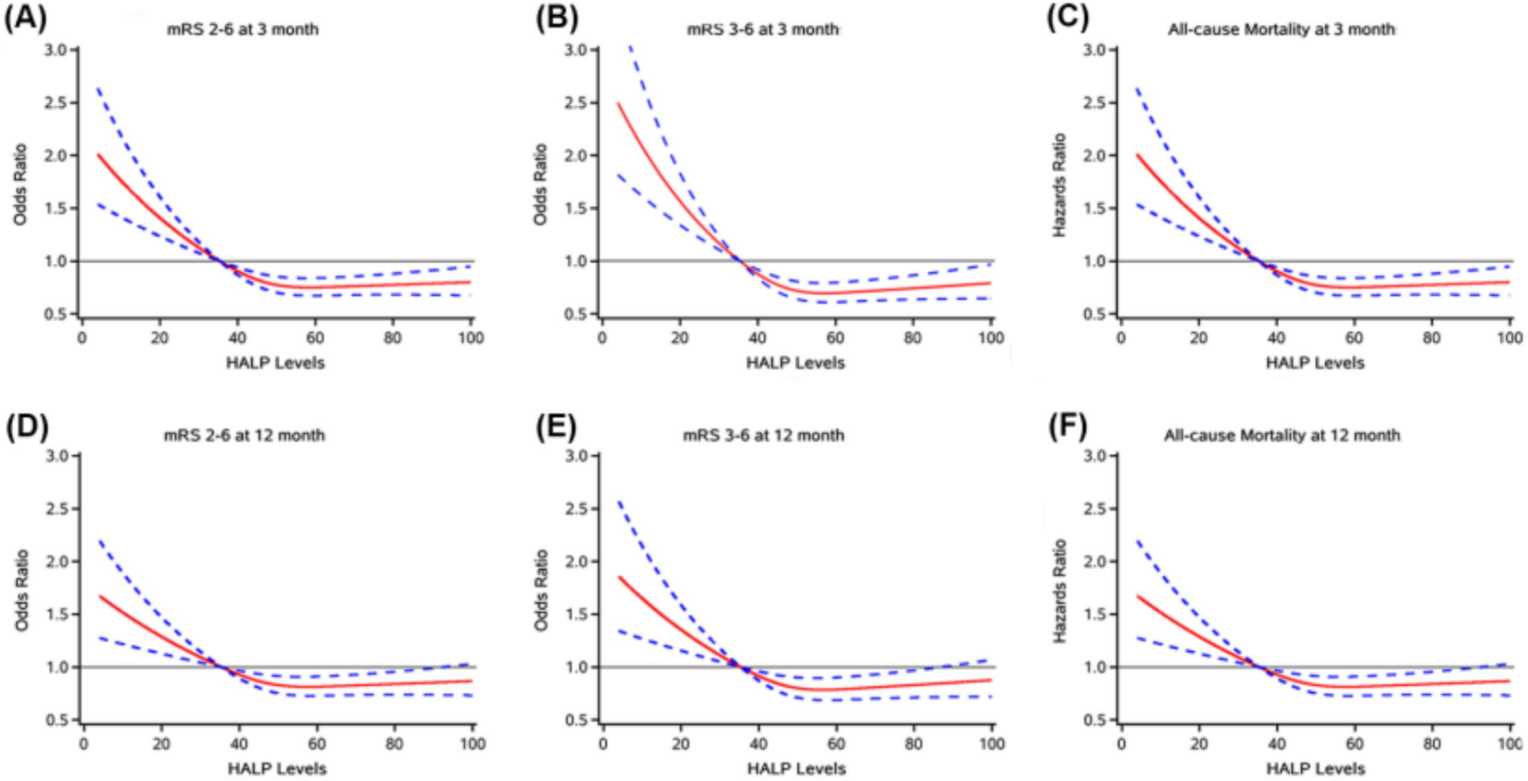
Restricted cubic splines showed the association between HALP score and poor functional outcomes (mrs2-6, mrs3-6, Mortality) at 3 months **(A, B, C)** and 12 months **(D, E, F)**. The red line indicates adjusted OR/HR, and the blue lines indicate the 95%Cl. Adjusted for age, gender, SBP, BMI, NIHSS score at admission, drinking and smoking, TOAST (LAA, CE, SAO, others), medical history (AIS or TIA, ICH, hypertension, coronary heart disease, dyslipidemia), in-hospital treatment (TPA, MT, antiplatelet, anticoagulant, antihypertensive, antihyperlipidemic, TCM).

At the 3-month follow-up, 563 patients (15.63%) had an mRS score of 3–6, and 1,113 patients (30.90%) had an mRS score of 2–6. At the 1-year follow-up, 538 patients (14.93%) had an mRS score of 3–6, and an mRS score of 2–6 occurred in 991 patients (27.50%). There was a significant change in the distribution of mRS scores according to HALP scores, with high HALP levels having a low rate of poor outcome and all-cause mortality (Figure 2). We present both unadjusted and adjusted correlations between HALP scores and clinical outcomes in Table 2. After adjusting for potential confounding variables, patients in the fourth quartile group with high HALP scores were significantly associated with a decreased risk of poor outcomes at 3 months compared to the first quartile group, with a lower adjusted OR of 0.53 (95% CI 0.40–0.72) for mRS scores 3–6 and 0.64 (95% CI 0.51–0.80) for mRS score 2–6. Similar trends were observed for outcomes at 1 year, the adjusted ORs in the fourth quartile group were 0.64 (95% CI 0.47–0.86) and 0.65 (95% CI 0.51–0.82) for mRS scores 3–6 and 2–6, respectively. Multivariable-adjusted spline regression models showed a non-linear relationship between the HALP scores and poor outcomes. At 3-month and 1-year follow-up, higher HALP scores was associated with a lower risk of poor outcomes and mRS scores of 3–6 and 2–6 OR steadily declined, showing L-type association (Figure 3).

**Table 2:** Association between HALP score and clinical outcomes.

	3-month follow-up			12-month follow-up		
Outcome	Events, *n* (%)	Unadjusted	adjusted	Events, *n* (%)	Unadjusted	adjusted
mRS 3 ~ 6	564 (15.65)			538 (14.93)		
Q1	211 (5.86)	Ref	Ref	184 (5.11)	Ref	Ref
Q2	143 (3.97)	0.62 (0.49–0.78)	0.69 (0.53–0.90)	143 (3.97)	0.73 (0.58–0.93)	0.90 (0.69–1.18)
Q3	117 (3.25)	0.49 (0.38–0.62)	0.56 (0.42–0.74)	122 (3.39)	0.61 (0.47–0.78)	0.76 (0.57–1.01)
Q4	93 (2.58)	0.38 (0.29–0.49)	0.53 (0.40–0.72)	89 (2.47)	0.43 (0.33–0.56)	0.64 (0.47–0.86)
*P* for trend	<0.001	<0.001	<0.001	<0.001	<0.001	0.002
mRS 2 ~ 6	1,113 (30.89)			991 (27.50)		
Q1	362 (10.05)	Ref	Ref	316 (8.77)	Ref	Ref
Q2	280 (7.77)	0.67 (0.55–0.81)	0.73 (0.59–0.91)	255 (7.08)	0.73 (0.60–0.89)	0.83 (0.67–1.04)
Q3	252 (6.99)	0.58 (0.47–0.70)	0.67 (0.54–0.84)	235 (6.52)	0.65 (0.53–0.80)	0.79 (0.63–0.99)
Q4	219 (6.08)	0.48 (0.39–0.58)	0.64 (0.51–0.80)	185 (5.13)	0.48 (0.39–0.59)	0.65 (0.51–0.82)
*P* for trend	<0.001	<0.001	<0.001	<0.001	0.003	<0.001
All-cause mortality	52 (1.44)			123 (3.41)		
Q1	23 (0.64)	Ref	Ref	54 (1.50)	Ref	Ref
Q2	11 (0.31)	0.48 (0.23–0.97)	0.61 (0.29–1.26)	30 (0.83)	0.55 (0.35–0.85)	0.69 (0.44–1.09)
Q3	13 (0.36)	0.56 (0.28–1.11)	0.75 (0.37–1.51)	26 (0.72)	0.47 (0.30–0.75)	0.61 (0.38–0.98)
Q4	5 (0.14)	0.22 (0.08–0.57)	0.35 (0.13–0.93)	13 (0.36)	0.23 (0.13–0.43)	0.34 (0.18–0.63)
*P* for trend	0.004	0.002	0.049	<0.001	<0.001	<0.001

Adjusted for age, gender, SBP, BMI, NIHSS score at admission, drinking and smoking, TOAST (LAA, CE, SAO, others), medical history (AIS or TIA, ICH, hypertension, coronary heart disease, dyslipidemia), in-hospital treatment (TPA, MT, antiplatelet, anticoagulant, antihypertensive, antihyperlipidemic, TCM). HALP, hemoglobin-albumin lymphocyte, and platelet scoring system; BMI, body mass index; SBP, systolic blood pressure; NIHSS, National Institute of Health Stroke Scale; mRS, modified Rankin Scale; AlS, acute ischemic stroke; TlA, transient ischemic attack; ICH, intracranial hemorrhage; TOAST, the Trail of Org 10,172 in Acute Stroke Treatment; LAA, large artery atherosclerosis; CE, cardioembolism; SAO, small artery occlusion; TPA, tissue plasminogen activator; MT, mechanical thrombectomy; TCM, traditional Chinese medicine.

**Figure 4:**
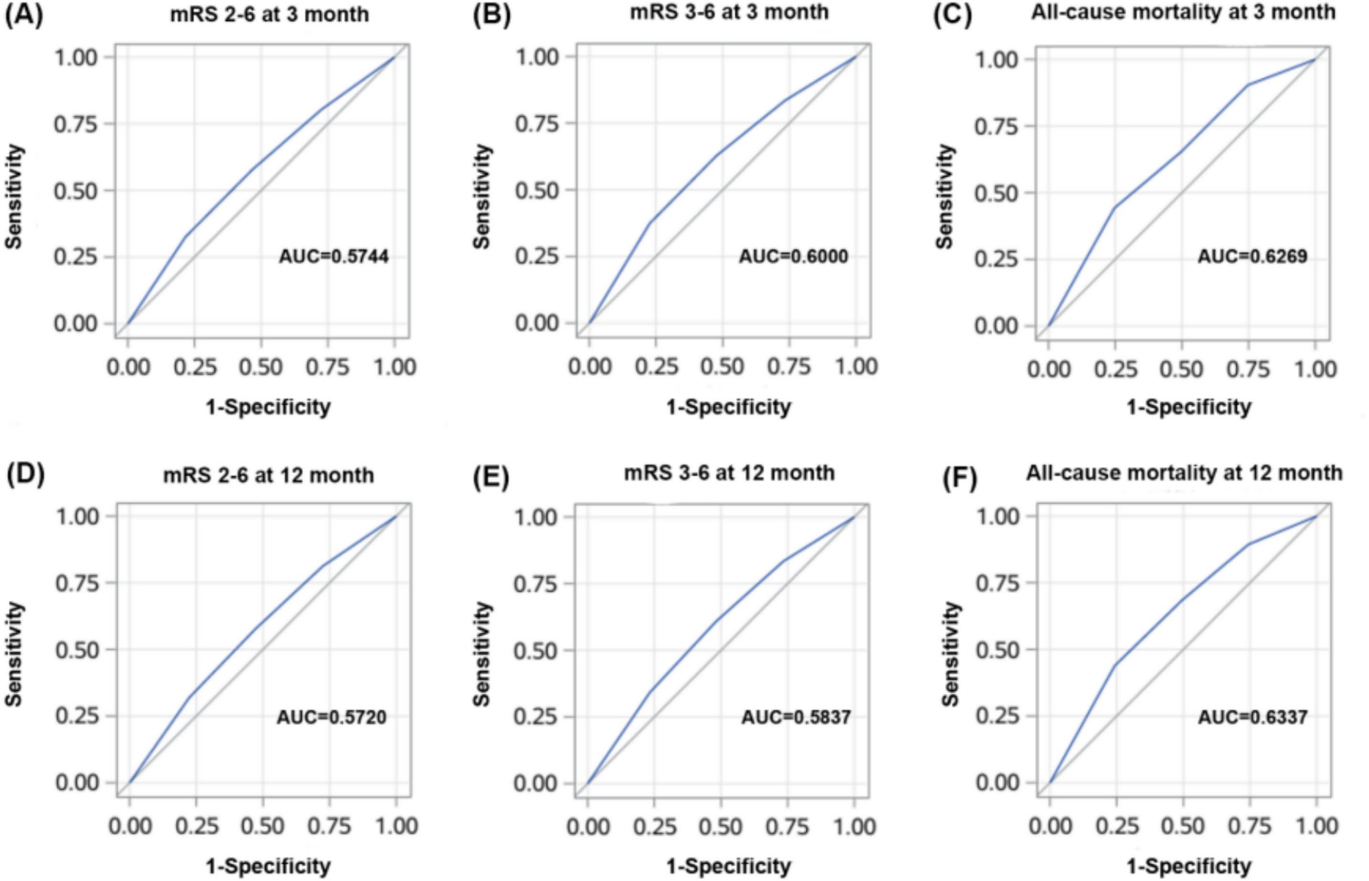
ROC curves of HALP score for poor clinical outcomes (mrs2-6, mrs3-6, mortality) at 3 months **(A, B, C)** and 12 months **(D, E, F)**.

The ROC curve of the HALP score for clinical outcomes has a good predictive value as shown in Figure 4 and the AUC values, cutoff values, *p*-values, sensitivity, and specificity presented in Table 3.

**Table 3:** Predictive values of HALP score for poor functional outcomes.

Outcome	AUC	Cutoff	OR (95%CI)	Sensitivity (%)	Specificity (%)
3 month follow-up					
mRS 2 ~ 6	0.5744	43.77	0.79 (0.74–0.84)	57.68	53.45
mRS 3 ~ 6	0.6000	43.77	0.72 (0.66–0.78)	62.76	52.38
Mortality	0.6269	27.71	0.65 (0.50–0.85)	44.23	75.30
12 month follow-up					
mRS 2 ~ 6	0.5720	53.06	0.79 (0.74–0.85)	57.62	52.91
mRS 3 ~ 6	0.5837	52.29	0.76 (0.70–0.83)	60.78	51.91
Mortality	0.6337	28.93	0.64 (0.54–0.76)	43.90	75.69

HALP, hemoglobin-albumin lymphocyte, and platelet scoring system; AUC, area under the curve; OR, odd ratios; mRS, modified Rankin Scale.

### Subgroup analysis

**Figure 5:**
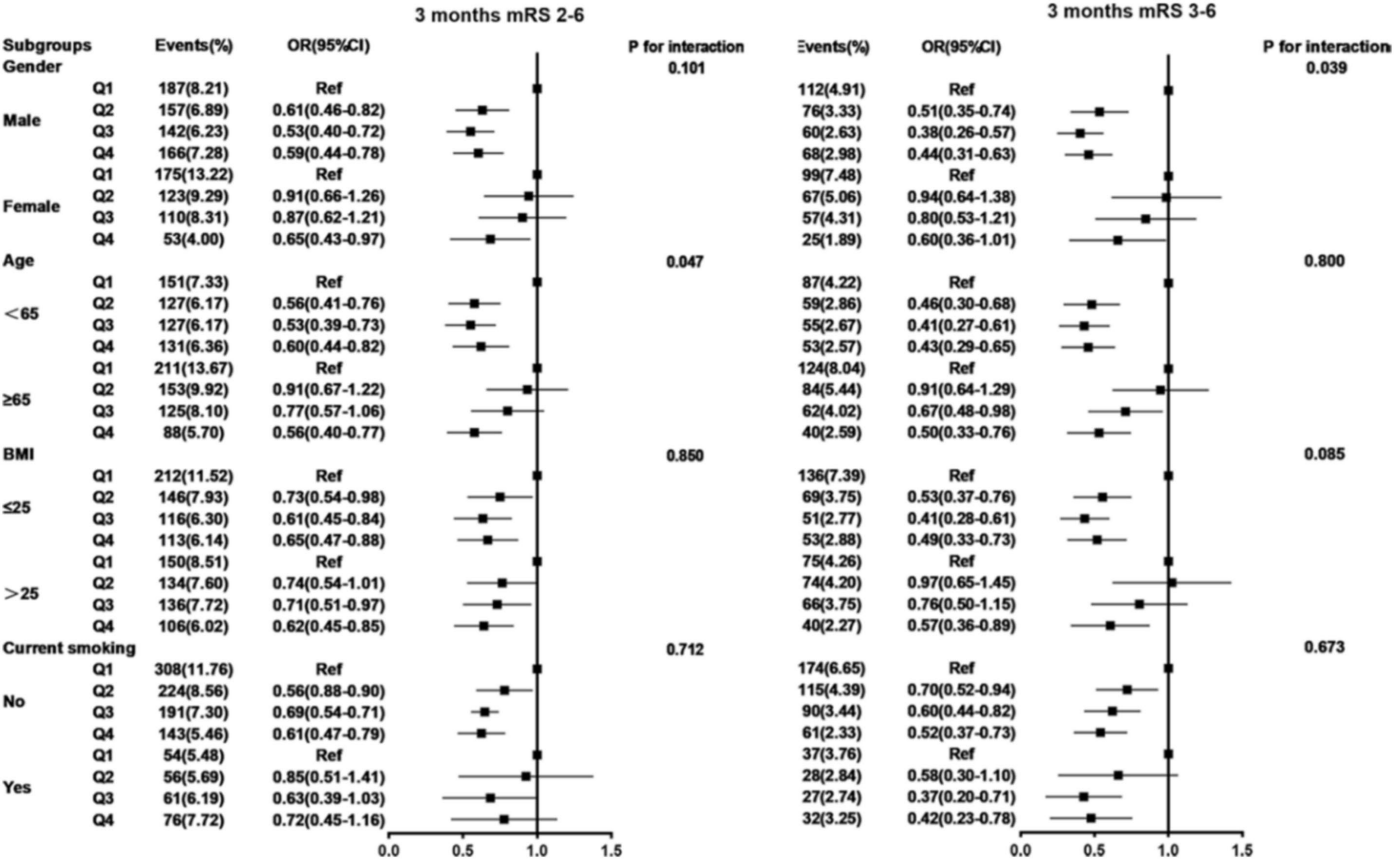
Subgroup analysis between HALP score and poor functional outcomes of patients with AIS and DM at 3 months follow-up. Adjusted for age, gender, SBP, BMI, NIHSS score at admission, drinking and smoking, TOAST (LAA, CE, SAO, others), medical history (AIS or TIA, ICH, hypertension, coronary heart disease, dyslipidemia), in-hospital treatment (TPA, MT, antiplatelet, anticoagulant, antihypertensive, antihyperlipidemic, TCM) except the stratified variables. HALP, hemoglobin-albumin lymphocyte, and platelet scoring system; BMI, body mass index; SBP, systolic blood pressure; NIHSS, National Institute of Health Stroke Scale; mRS, modified Rankin Scale; AlS, acute ischemic stroke; TlA, transient ischemic attack; ICH, intracranial hemorrhage; TOAST, the Trail of Org 10,172 in Acute Stroke Treatment; LAA, large artery atherosclerosis; CE, cardioembolism; SAO, small artery occlusion; TPA, tissue plasminogen activator; MT, mechanical thrombectomy; TCM, traditional Chinese medicine.

**Figure 6:**
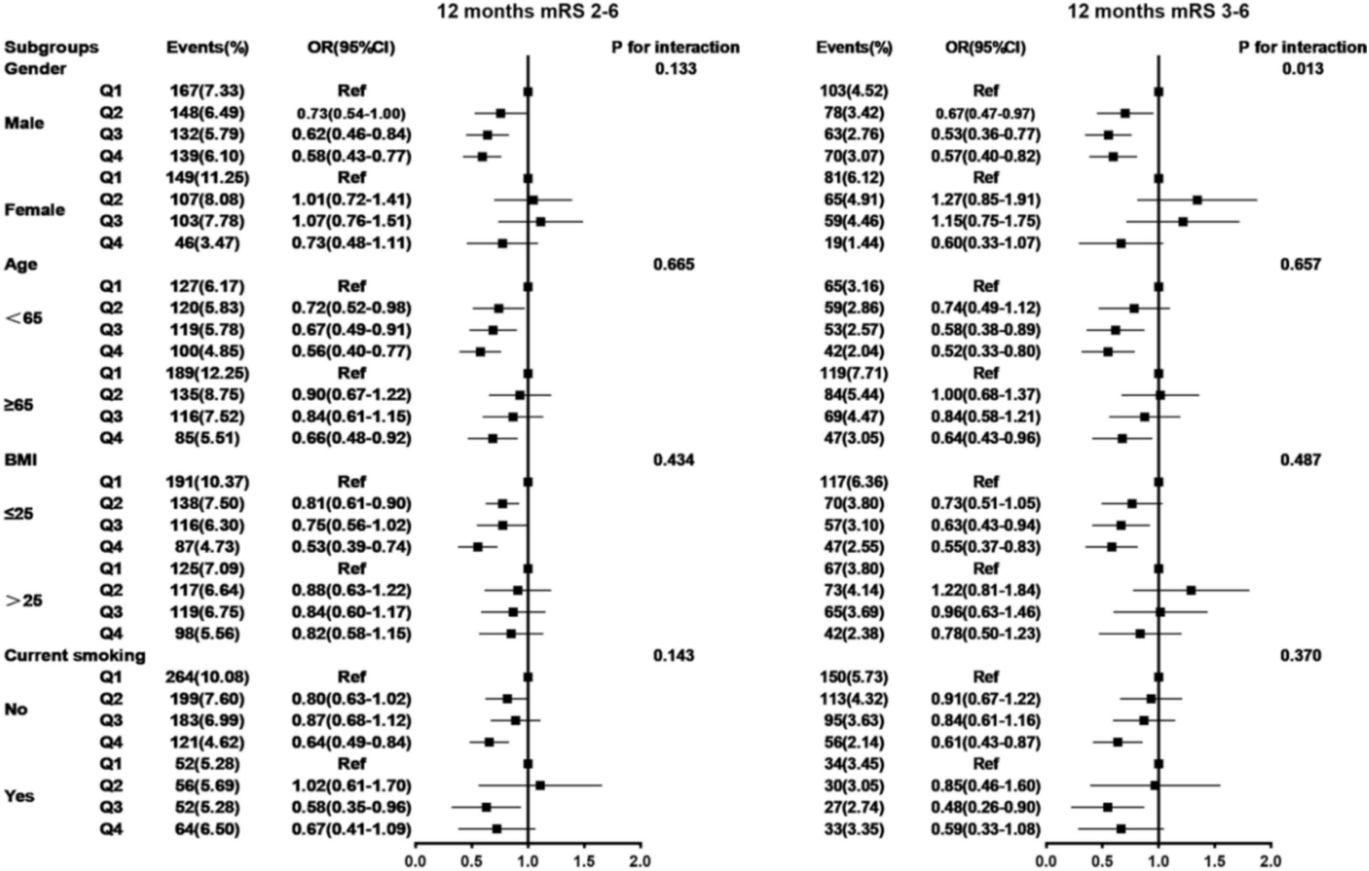
Subgroup analysis between HALP score and poor functional outcomes of patients with AIS and DM at 12 months follow-up. Adjusted for age, gender, SBP, BMI, NIHSS score at admission, drinking and smoking, TOAST (LAA, CE, SAO, others), medical history (AIS or TIA, ICH, hypertension, coronary heart disease, dyslipidemia), in-hospital treatment (TPA, MT, antiplatelet, anticoagulant, antihypertensive, antihyperlipidemic, TCM) except the stratified variables. HALP, hemoglobin-albumin lymphocyte, and platelet scoring system; BMI, body mass index; SBP, systolic blood pressure; NIHSS, National Institute of Health Stroke Scale; mRS, modified Rankin Scale; AlS, acute ischemic stroke; TlA, transient ischemic attack; ICH, intracranial hemorrhage; TOAST, the Trail of Org 10,172 in Acute Stroke Treatment; LAA, large artery atherosclerosis; CE, cardioembolism; SAO, small artery occlusion; TPA, tissue plasminogen activator; MT, mechanical thrombectomy; TCM, traditional Chinese medicine.

Subgroup analyses were conducted and stratified by age, gender, BMI, and current smoking. The results are shown in Table 4 and Figures 5, 6, indicating minimal significant interaction between HALP score and the stratified variables, except in the case of gender with 3-month and 1-year mRS score of 3–6 (*p*-value for interaction: 0.039 and 0.013) which suggests that the correlation between HALP and poor functional prognosis differs by gender.

**Table 4:** Subgroup analysis.

Subgroup	Adjusted OR (95%CI)				*P*-value for interaction
	Q1	Q2	Q3	Q4	
3 months MRS 2-6					
Gender					0.101
Male	Ref	0.61(0.46–0.82)	0.53(0.40–0.72)	0.59(0.44–0.78)	
Female	Ref	0.91(0.66–1.26)	0.87(0.62–1.21)	0.65(0.43–0.97)	
Age					0.047
<65	Ref	0.56(0.41–0.76)	0.53(0.39–0.73)	0.60(0.44–0.82)	
≥65	Ref	0.91(0.67–1.22)	0.77(0.57–1.06)	0.56(0.40–0.77)	
BMI					0.850
≤25	Ref	0.73(0.54–0.98)	0.61(0.45–0.84)	0.65(0.47–0.88)	
>25	Ref	0.74(0.54–1.01)	0.71(0.51–0.97)	0.62(0.45–0.85)	
Current smoking					0.712
No	Ref	0.56(0.88–0.90)	0.69(0.54–0.71)	0.61(0.47–0.79)	
Yes	Ref	0.85(0.51–1.41)	0.63(0.39–1.03)	0.72(0.45–1.16)	
3 months MRS 3-6					
Gender					0.039
Male	Ref	0.51(0.35–0.74)	0.38(0.26–0.57)	0.44(0.31–0.63)	
Female	Ref	0.94(0.64–1.38)	0.80(0.53–1.21)	0.60(0.36–1.01)	
Age					0.800
<65	Ref	0.46(0.30–0.68)	0.41(0.27–0.61)	0.43(0.29–0.65)	
≥65	Ref	0.91(0.64–1.29)	0.67(0.48–0.98)	0.50(0.33–0.76)	
BMI					0.085
≤25	Ref	0.53(0.37–0.76)	0.41(0.28–0.61)	0.49(0.33–0.73)	
>25	Ref	0.97(0.65–1.45)	0.76(0.50–1.15)	0.57(0.36–0.89)	
Current smoking					0.673
No	Ref	0.70(0.52–0.94)	0.60(0.44–0.82)	0.52(0.37–0.73)	
yes	Ref	0.58(0.30–1.10)	0.37(0.20–0.71)	0.42(0.23–0.78)	
3 months mortality					
Gender					0.015
Male	Ref	0.33(0.13–0.84)	0.20(0.06–0.60)	0.28(0.10–0.80)	
Female	Ref	1.66(0.44–6.26)	2.93(0.91–9.44)	0.00(0.00–0.00)	
Age					0.126
<65	Ref	0.72(0.22–2.73)	0.16(0.02–1.39)	0.32(0.06–1.72)	
≥65	Ref	0.38(0.14–1.05)	1.08(0.50–2.33)	0.28(0.08–0.97)	
BMI					0.493
≤25	Ref	0.39(0.12–1.22)	0.49(0.17–1.41)	0.31(0.08–1.10)	
>25	Ref	1.11(0.39–3.23)	1.25(0.44–3.54)	0.43(0.09–2.15)	
Current smoking					0.323
No	Ref	0.80(0.36–1.78)	0.93(0.42–2.05)	0.23(0.05–1.03)	
Yes	Ref	0.23(0.02–2.21)	0.40(0.07–2.27)	0.46(0.10–2.21)	
12 months MRS 2-6					
Gender					0.133
Male	Ref	0.73(0.54–1.00)	0.62(0.46–0.84)	0.58(0.43–0.77)	
Female	Ref	1.01(0.72–1.41)	1.07(0.76–1.51)	0.73(0.48–1.11)	
Age					0.665
<65	Ref	0.72(0.52–0.98)	0.67(0.49–0.91)	0.56(0.40–0.77)	
≥65	Ref	0.90(0.67–1.22)	0.84(0.61–1.15)	0.66(0.48–0.92)	
BMI					0.434
≤25	Ref	0.81(0.61–0.90)	0.75(0.56–1.02)	0.53(0.39–0.74)	
>25	Ref	0.88(0.63–1.22)	0.84(0.60–1.17)	0.82(0.58–1.15)	
Current smoking					0.143
No	Ref	0.80(0.63–1.02)	0.87(0.68–1.12)	0.64(0.49–0.84)	
Yes	Ref	1.02(0.61–1.70)	0.58(0.35–0.96)	0.67(0.41–1.09)	
12 months MRS 3-6					
Gender					0.013
Male	Ref	0.67(0.47–0.97)	0.53(0.36–0.77)	0.57(0.40–0.82)	
Female	Ref	1.27(0.85–1.91)	1.15(0.75–1.75)	0.60(0.33–1.07)	
Age					0.657
<65	Ref	0.74(0.49–1.12)	0.58(0.38–0.89)	0.52(0.33–0.80)	
≥65	Ref	1.00(0.68–1.37)	0.84(0.58–1.21)	0.64(0.43–0.96)	
BMI					0.487
≤25	Ref	0.73(0.51–1.05)	0.63(0.43–0.94)	0.55(0.37–0.83)	
>25	Ref	1.22(0.81–1.84)	0.96(0.63–1.46)	0.78(0.50–1.23)	
Current smoking					0.370
No	Ref	0.91(0.67–1.22)	0.84(0.61–1.16)	0.61(0.43–0.87)	
Yes	Ref	0.85(0.46–1.60)	0.48(0.26–0.90)	0.59(0.33–1.08)	
12 months mortality					
Gender					0.072
Male	Ref	0.54(0.30–0.96)	0.32(0.17–0.63)	0.26(0.12–0.53)	
Female	Ref	1.01(0.46–2.20)	1.31(0.63–2.70)	0.49(0.14–1.68)	
Age					0.783
<65	Ref	0.57(0.25–1.28)	0.44(0.18–1.05)	0.30(0.11–0.83)	
≥65	Ref	0.69(0.40–1.20)	0.72(0.41–1.27)	0.33(0.15–0.71)	
BMI					0.529
≤25	Ref	0.59(0.32–1.11)	0.46(0.23–0.93)	0.23(0.09–0.55)	
>25	Ref	0.96(0.48–1.96)	0.86(0.42–1.75)	0.54(0.22–1.33)	
Current smoking					0.345
No	Ref	0.85(0.52–1.39)	0.69(0.41–1.18)	0.39(0.19–0.81)	
Yes	Ref	0.26(0.07–0.96)	0.36(0.12–1.08)	0.17(0.05–0.63)	

Adjusted for age, gender, SBP, BMI, NIHSS score at admission, drinking and smoking, TOAST (LAA, CE, SAO, others), medical history (AIS or TIA, ICH, hypertension, coronary heart disease, dyslipidemia), in-hospital treatment (TPA, MT, antiplatelet, anticoagulant, antihypertensive, antihyperlipidemic, TCM) except the stratified variables. OR, odd ratios; mRS, modified Rankin Scale; BMI, body mass index.

## Discussion

The primary finding of the study in the CNSR-III was that a reduced HALP score is significantly associated with an increased risk of poor functional prognosis and all-cause mortality in AIS patients during 3-month and 1-year follow-ups.

Inflammation is commonly regarded as a precursor to atherosclerosis and diabetes (18). Furthermore, type 2 DM constitutes a disorder characterized by nutritional and metabolic dysfunctions. HALP score can comprehensively and effectively reflect the inflammatory-nutritional status of patients with AIS and diabetes. Ischemic brain tissue activates immune cells, promoting their migration to ischemic sites through the release of pro-inflammatory chemokines (19). A local inflammatory response stimulates vasoconstriction and triggers thrombosis and platelets contribute to this process through adhesion, release responses, and aggregation. Studies have demonstrated a clear association between platelet count and the poor prognosis and recurrence of ischemic stroke (20). Lymphocytes play a role in regulating the inflammatory response and promoting tissue repair. A higher lymphocyte count can mitigate the enlargement of the infarct area in ischemic stroke, improving neurological function and outcomes (21). Albumin has neuroprotective and repair functions and also inhibits oxidative stress, inflammatory response, and thrombosis (22). Hemoglobin has an oxygen-carrying capacity which helps reduce brain tissue damage in the penumbra. Consequently, low hemoglobin and hematocrit levels are significant contributors to poor prognosis and mortality following AIS (23). The HALP score is a cost-effective, simple, and accessible method to assess the inflammatory-nutritional status of AIS patients with diabetes in hospital, aiding clinicians in evaluating prognosis and formulating appropriate treatment plans. Wang N et al. evaluated the lymphocyte-to-monocyte ratio (LMR), neutrophil-to-lymphocyte ratio (NLR), eosinophil-to-lymphocyte ratio (ELR), basophil-to-lymphocyte ratio (BLR), platelet-to-lymphocyte ratio (PLR), as well as the HALP, the predictive value of these indicators and constructed predictive models, and found that the inclusion of HALP individually further enhanced the predictive efficacy in the study of acute exacerbations of chronic obstructive pulmonary disease (24). In addition, a cohort study of 411 patients with early-stage breast cancer found that HALP had a higher predictive value than NLR, PLR, LMR, and prognostic nutritional index (PNI). HALP is an independent risk factor for early-stage breast cancer, which is significantly associated with recurrence-free survival and can be used as an effective predictor of tumor recurrence or metastasis (25).

Previous research indicated the HALP score is mostly associated with the prognosis of lung cancer, kidney cancer, gastric cancer, and other diseases (26). Guc et al. (27) demonstrated that the HALP score serves as a simple, cost-effective, and valuable predictor of overall survival rate and prognosis in non-small-cell lung cancer. Recent investigations have revealed that the HALP score holds significant clinical value for cardiovascular and cerebrovascular diseases. Zheng et al. (14) conducted a study on a retrospective cohort within the National Health and Nutrition Examination Survey (NHANES) database, finding a negative association between the HALP score and all-cause mortality from coronary heart disease. Within the nervous system, a significant association was observed between HALP scores and cognitive function. A low HALP score increases the risk of cognitive impairment after stroke (28, 29). Tian et al. (30) discovered that an elevated HALP score was associated with a decreased risk of recurrent stroke and poor prognosis. Many previous studies were characterized by small sample sizes or a lack of long-term follow-up data. Our study focused on a population primarily consisting of stroke patients with type 2 diabetes, and our findings align with prior research. Additionally, our study featured a large sample size and a follow-up period of 1 year. However, there is limited information on the relationship between HALP score and prognosis and all-cause mortality. Due to the role of HALP score in the prognosis of patients with various types of cancer, we explored and demonstrated an inverse relationship between HALP score and long-term prognosis and all-cause mortality in AIS. The potential value of the HALP score for all-cause mortality in patients with AIS has an AUC slightly higher than that of long-term prognostic indicators. Further analysis showed that HALP scores were non-linearly associated with prognosis and all-cause mortality in AIS and the primary cause of which may be related to high levels of hemoglobin and lymphocyte counts. A cohort study of 3,481 adults showed an L-shaped relationship between hemoglobin levels and poor prognosis (31). In addition, hemoglobin is an independent risk factor for poor prognosis and mortality after ischemic stroke and is strongly associated with low and further declining Hb levels (32). On the other hand, Kim et al. (21) included 779 patients with AIS in their study and found that lower lymphocyte counts with less improvement were strongly associated with a poorer prognosis at 3 months by promoting increased infarct volume and deterioration of neurologic function. Lymphocytes play a crucial role in eliminating inflammation and repairing nerves.

In this study, the results of subgroup analysis suggested an interaction between gender and HALP score. The reason may be the difference in HGB levels between males and females. The results of a health survey from several cities in Korea showed that the prevalence of anemia was 2.98% in men and 8.56% in women, and the prevalence of severe anemia was 0.23% in men and 1.51% in women (33). There were significant differences between genders, which may be the main reason for the gender interaction.

The primary strength of this study lies in its design as a multicenter prospective registry study with a large sample size and a long-term follow-up record, thus providing sufficient impetus for the statistical analysis. However, this study also has some limitations. First, there may be selection bias due to the exclusion of patients with incomplete data or missing follow-up information on baseline hemoglobin, albumin, lymphocyte, and platelet counts. Second, since all patients were from China, these findings should be cautiously extrapolated to other populations. Additional prospective studies involving diverse populations are necessary to validate our findings. Finally, owing to the study design constraints, it was limited to evaluating HALP scores at admission without tracking their dynamic changes at different stages or collecting data on biochemical physiological parameters and medications before acute stroke onset or during the follow-up period.

## Conclusion

This study demonstrated that lower HALP score levels were associated with poorer functional outcomes and all-cause mortality at the 3-month and 1-year follow-up in AIS patients with type 2 diabetes. HALP may be a potential therapeutic target for AIS patients with type 2 diabetes mellitus.

## Data Availability

The original contributions presented in the study are included in the article/supplementary material, further inquiries can be directed to the corresponding authors.
